# Is Angiosome-Targeted Angioplasty Effective for Limb Salvage and Wound Healing in Diabetic Foot? : A Meta-Analysis

**DOI:** 10.1371/journal.pone.0159523

**Published:** 2016-07-21

**Authors:** Kum Ju Chae, Jin Yong Shin

**Affiliations:** 1 Department of Radiology, Chonbuk National University Hospital, Jeonju, Korea; 2 Department of Plastic and Reconstructive Surgery, Chonbuk National University Hospital, Jeonju, Korea; University of Insubria, ITALY

## Abstract

**Purpose:**

Given that the efficacy of employing angiosome-targeted angioplasty in the treatment of diabetic foot remains controversial, this study was conducted to examine its efficacy.

**Methods:**

We performed a systematic literature review and meta-analysis using core databases, extracting the treatment modality of angiosome-targeted angioplasty as the predictor variable, and limb salvage, wound healing, and revision rate as the outcome variables. We used the Newcastle-Ottawa Scale to assess the study quality, along with the Cochrane Risk of Bias Tool. We evaluated publication bias using a funnel plot.

**Results:**

The search strategy identified 518 publications. After screening these, we selected four articles for review. The meta-analysis revealed that overall limb salvage and wound healing rates were significantly higher (Odds ratio = 2.209, 3.290, p = 0.001, p<0.001) in patients who received angiosome-targeted angioplasty than in those who received nonangiosome-targeted angioplasty. The revision rate between the angiosome and nonangiosome groups was not significantly different (Odds ratio = 0.747, p = 0.314).

**Conclusion:**

Although a further randomized controlled trial is required for confirmation, angiosome-targeted angioplasty in diabetic foot was more effective than nonangiosome-targeted angioplasty with respect to wound healing and limb salvage.

## Introduction

Peripheral arterial disease is present in up to 50% of patients with diabetic foot [1], and those patients face several difficulties not commonly found in the case of general ischemic limbs. Poor vascular connectivity between angiosomes in a diabetic foot can result in treatment failure for ulcers [2]. Because of reduced blood flow to microvascular beds, the Trans-Atlantic Inter-Society Consensus Document on the Management of Peripheral Arterial Disease II guidelines state that the amputation rate is higher in diabetic patients than in other patients [3]. Furthermore, patients with diabetic feet have prolonged tissue healing time because of impaired host defense mechanisms against infections [4–6]. These findings delineate the challenges that doctors face when managing diabetic foot complicated by arterial occlusive disease.

To overcome those difficulties, researchers have developed several procedures and surgeries for revascularization of diabetic feet with arterial occlusive disease. Angioplasty using the angiosome concept is the most recent intervention. The angiosome concept, first introduced by Taylor and Paler [7] approximately two decades ago, is considered an important factor in wound healing [7, 8].]. An angiosome is a unit of tissue supplied by a source artery via a three-dimensional network of vessels [7]. Several studies have reported outstanding revascularization outcomes upon using the angiosome concept in treating diabetic feet [9, 10].

However, the use of the angiosome concept is associated with some limitations and controversy in regard to diabetic foot treatment. Applying this concept is not always appropriate because of infection, severe arterial disease, or the absence of a source artery for the lesion [2, 11], and most vascular surgeons believe that a proper bypass graft is sufficient to supply the entire foot, irrespective of the angiosome associated with the lesion [2]. The purpose of this study was to further evaluate the efficacy of using the angiosome concept when performing angioplasty on diabetic foot. Hence, our meta-analysis examined the outcomes in terms of wound healing, limb salvage, and revision rate when angioplasty for the diabetic foot was performed in a manner consistent with use of the angiosome concept.

## Materials and Methods

### Institutional Review Board

Institutional Review Board approval is not required for a meta-analysis.

### Literature search and selection

We investigated eligible articles using the PubMed, EMBASE, and Cochrane databases for all studies related to revascularization associated with arterial occlusive disease in diabetic feet prior to February 2016. We used the Medical subject headings keywords *angiosome*, *diabetic foot*, and *revascularization* because all core databases use it. We also investigated all relevant articles to identify additional studies.

We included prospective and retrospective observational studies that met the following criteria: 1) a full-length article that provided sufficient data to enable evaluation of the angiosome concept in diabetic foot; 2) a brief statement addressing treatment modalities, revascularized vessels, and outcome variables; and 3) inclusion of a comparison group of diabetic feet treated with nonangiosome-targeted revascularization. The exclusion criteria were as follows: 1) incomplete data; 2) review or case study articles; 3) abstract-only studies; 4) articles describing fewer than 10 cases; or 5) articles with overlapping authors.

### Data extraction

The predictor variables were angiosome- or nonangiosome-targeted revascularization procedures applied to ischemic limbs with diabetic feet. The outcome variables were limb salvage rate, complete wound healing, and revision rate.

### Assessment of methodological quality

We assessed the methodological quality of the nonrandomized studies selected using the Newcastle-Ottawa Scale (NOS). The NOS is categorized into three parameters: selection of the study population, comparability of the groups, and ascertainment of the exposure or outcome. Each parameter consists of subcategorized questions [12, 13]. Two of the authors independently evaluated the methodological quality of the enrolled studies in our meta-analysis.

### Statistical Evaluation

We used the Comprehensive Meta-Analysis software (version 3.3.070, Biostat Inc.) for this meta-analysis. We calculated the limb salvage and wound healing rates, and assessed the heterogeneity of each study using the *I*^*2*^ test, which measures the percentage of heterogeneity across studies [14]. *I*^*2*^ was calculated as follows: *I*^*2*^ (%) = 100 × (*Q*-*df*)/*Q*, where *Q* is Cochrane’s heterogeneity statistic and *df* is the number of degrees of freedom. I^2^ statistics with values of 25%, 50%, and 75% mean low, moderate, and high heterogeneity, respectively. We then computed the 95% confidence interval (CI) of each treatment modality using random and fixed effects models. We confirmed those results using the *I*^*2*^ test, with significance set at *P* less than 0.05. We provided forest plots to describe study outcomes and funnel plots to assess publication bias.

## Results

### Study characteristics

**Fig 1:**
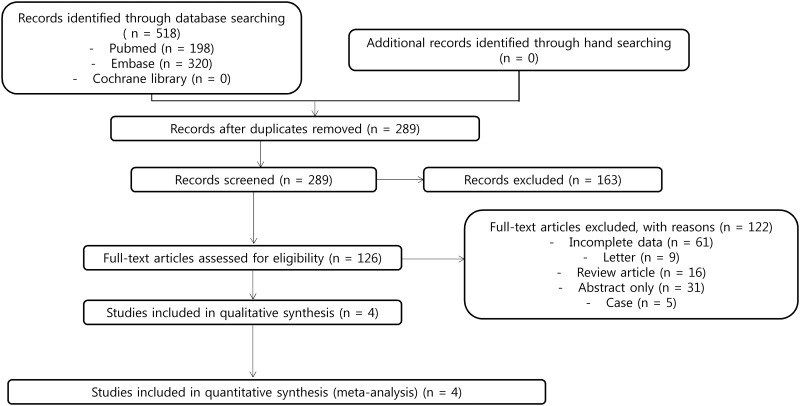
Flow diagram for identification of relevant studies.

Fig 1 shows a flow diagram of how we screened candidate studies. Database searches identified 518 publications that potentially met the study criteria, from which 229 were eliminated as duplicates. In the screening process, a review of titles and abstracts excluded 163 studies that did not meet the inclusion criteria. We reviewed the remaining 126 articles for eligibility by reviewing the full text. Reasons for study exclusion during the final screen were as follows: review articles (*n* = 16), incomplete data (*n* = 61), abstract only (n = 31), letter (*n* = 9), or case report (*n* = 5) (S1 Fig). We included the remaining four nonrandomized studies in the final analysis.

The four studies [15–18] included 727 patients with 881 diabetic feet. We identified these studies on the basis of their inclusion of two different treatment modalities, angiosome-targeted (direct) angioplasty and nonangiosome (indirect) angioplasty. The clinical characteristics of the patients in these studies are shown in Tables 1 and 2. These studies were retrospective, written in English, and published between 2011 and 2014. Patients enrolled in the studies were diagnosed with ischemic ulcers in diabetic feet. In all the studies, if revascularization targeted the direct tributary artery feeding the skin ulcer territory, it was defined as angiosome-targeted angioplasty, while nonangiosome-targeted angioplasty was defined as angioplasty improving flow in the ulcerated area via collateral vessels. In three of the studies [16–18], limb salvage and wound healing were noted 1 or 2 years after angioplasty; the remaining study [15] did not describe outcomes over time. The mean NOS score for the studies was 8 stars (Table 3).

**Table 1:** Clinical data of included studies.

Studies	Study design	Total No. of patients(limbs)	Age	Duration of follow up	Location, laguage
**Alexandrescu et al., 2011**	Retrospective study	208 (232)	Mean 74.3 (42–97 ranged)	Mean 38.6 months (1–68 ranged)	Belgium (English)
**Soderstrom et al., 2013**	Retrospective study	226 (250)	Mean 71.2 ± 11.8	At least 12months	Finland (English)
**Acin et al., 2014**	Retrospective study	92 (101)	Mean 72 (64–77 ranged)	Median 19 months (9–38 ranged)	Spain (English)
**Fossaceca et al., 2013**	Retrospective study	201 (298)	Mean 75.5 ± 9.5	Mean 17.5 ± 12 months	Italy (English)

**Table 2:** Clinical data of included studies.

Studies	Angiosome-targeted angioplasty				Non-angiosome-targeted angioplasty			
	Limb salvage	Wound healing	Revision	Treated vessels (ATA, PTA, PER)	Limb salvage	Wound healing	Revision	Treated vessels (ATA, PTA, PER)
**Alexandrescu et al., 2011**	130/134	106/134	*	34/134	83/98	54/98	*	22/98
				91/134				64/98
				9/134				12/98
**Soderstrom et al., 2013**	104/121	87/121	18/121	69/121	99/129	56/129	21/129	73/129
				57/121				24/129
				29/121				62/129
**Acin et al., 2014**	41/46	30/46	*	37/46	29/39	16/39	*	28/39
				39/46				38/39
				7/46				3/39
**Fossaceca et al., 2013**	151/167	163/167	16/167	*	31/34	29/34	6/34	*

* No data

**Table 3:** Methdological quality of included studies measured by Newcastle-Ottawa scale.

Studies	Selection	Comparability	Exposure or outcome	Total
**Alexandrescu et al., 2011**	☆☆☆☆	☆☆	☆☆	8
**Soderstrom et al., 2013**	☆☆☆☆	☆☆	☆	7
**Acin et al., 2014**	☆☆☆☆	☆☆	☆☆☆	9
**Fossaceca et al., 2013**	☆☆☆☆	☆☆	☆☆	8

In all included studies, wounds were treated with standardized approach. Local treatment such as early debridement of devitalized tissues, abscess drainage with antibiotic therapy, wet dressings and minor amputation were performed. If primary closure was not possible, possible skin graft or flap surgeries were considered.

### Meta-analysis of enrolled studies

**Fig 2:**
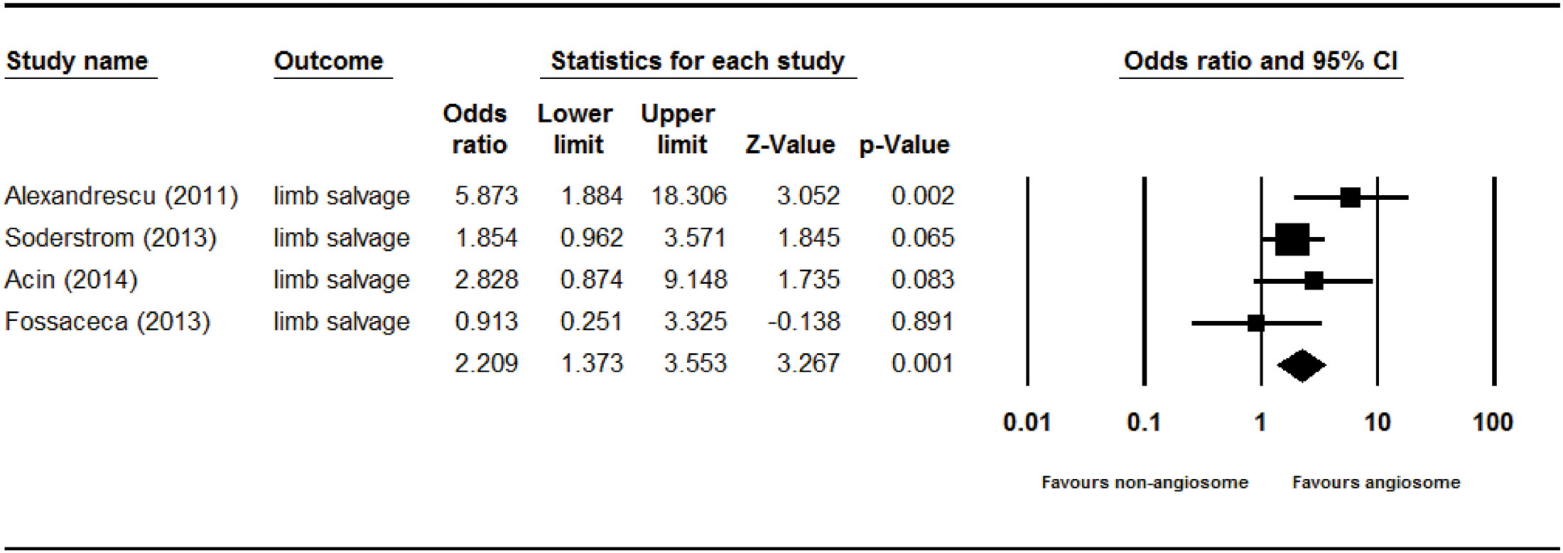
Forest plot of limb salvage rate of angiosome- and non-angiosome-targeted angioplasty. Heterogeneity: *χ*^2^ = 5.082, df = 3 (P = 0.166); I^2^ = 40.964%. Test for overall effect: Z = 3.267 (P = 0.001).

**Fig 3:**
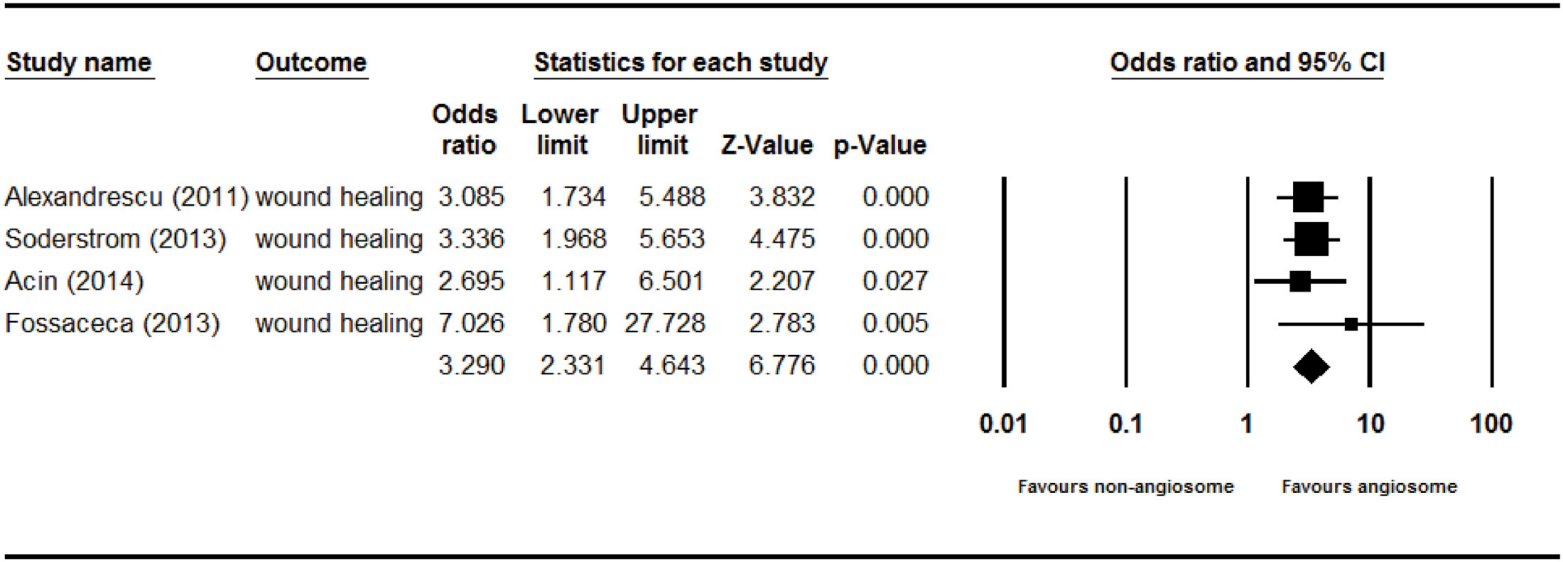
Forest plot of wound healing rate of angiosome- and non-angiosome-targeted angioplasty. Heterogeneity: *χ*^2^ = 1.421, df = 3 (P = 0.701); I^2^ = 0.000%. Test for overall effect: Z = 6.776 (P < 0.001).

**Fig 4:**
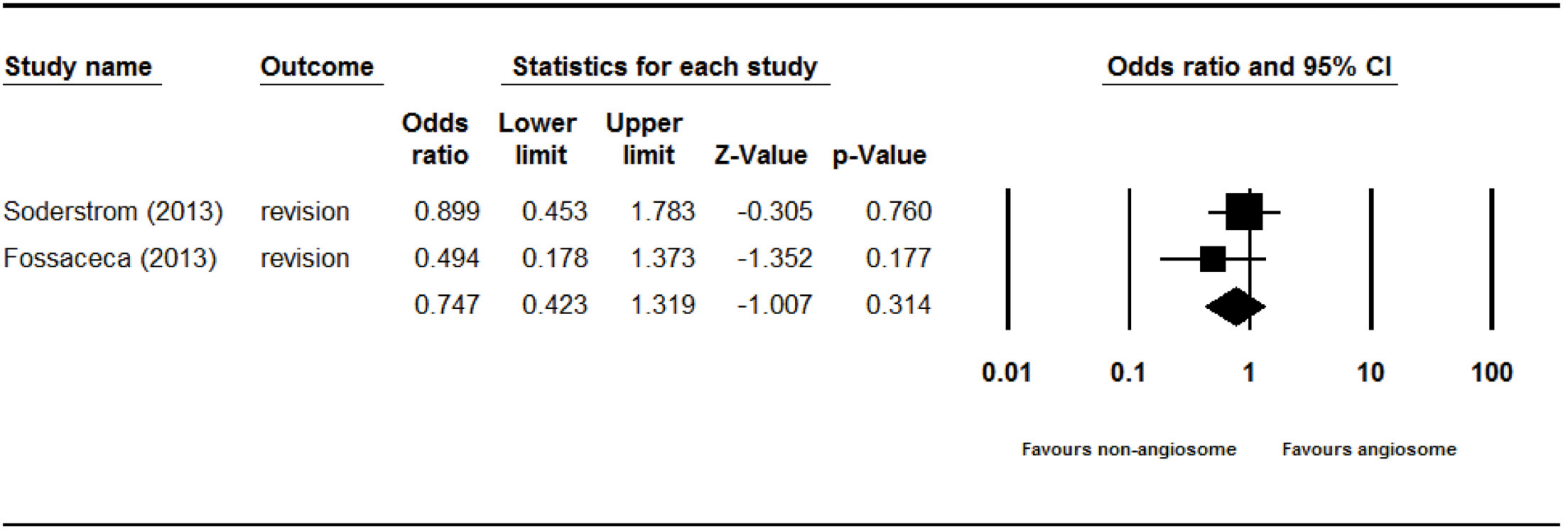
Forest plot of revision rate of angiosome- and non-angiosome-targeted angioplasty. Heterogeneity: *χ*^2^ = 0.907, df = 1 (P = 0.341); I^2^ = 0.000%. Test for overall effect: Z = −1.007 (P = 0.314).

The overall limb salvage rate of angiosome-targeted angioplasty of an ischemic limb in diabetic patients was significantly higher than that of nonangiosome-targeted angioplasty (odds ratio [OR] = 2.209, 95% CI: 1.373–3.553, p = 0.001) in a fixed effect model–based meta-analysis of the four studies (Fig 2). The overall wound healing rate of angiosome-targeted angioplasty was more favorable than that of nonangiosome-targeted angioplasty (OR = 3.290, 95% CI: 2.331–4.643, p = <0.001) in a fixed effect model–based meta-analysis of the four studies (Fig 3). The overall revision rates of angiosome-targeted angioplasty and nonangiosome-targeted angioplasty of an ischemic limb in diabetic patients were not significantly different (OR = 0.747, 95% CI: 0.423–1.319, p = 0.314) in a fixed effect model–based meta-analysis of two studies (Fig 4).

### Publication bias

**Fig 5:**
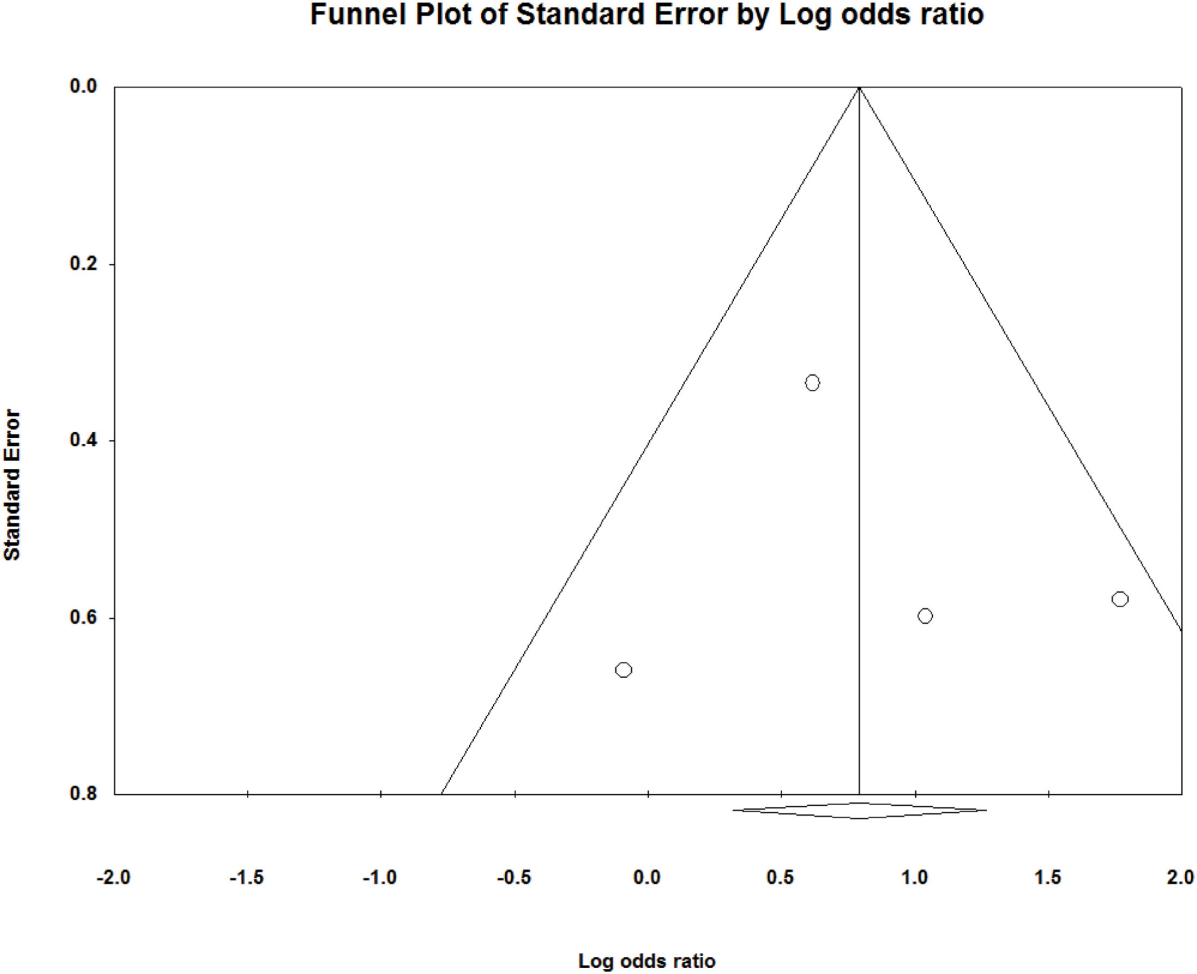
Funnel plot for publication bias in limb salvage rate.

**Fig 6:**
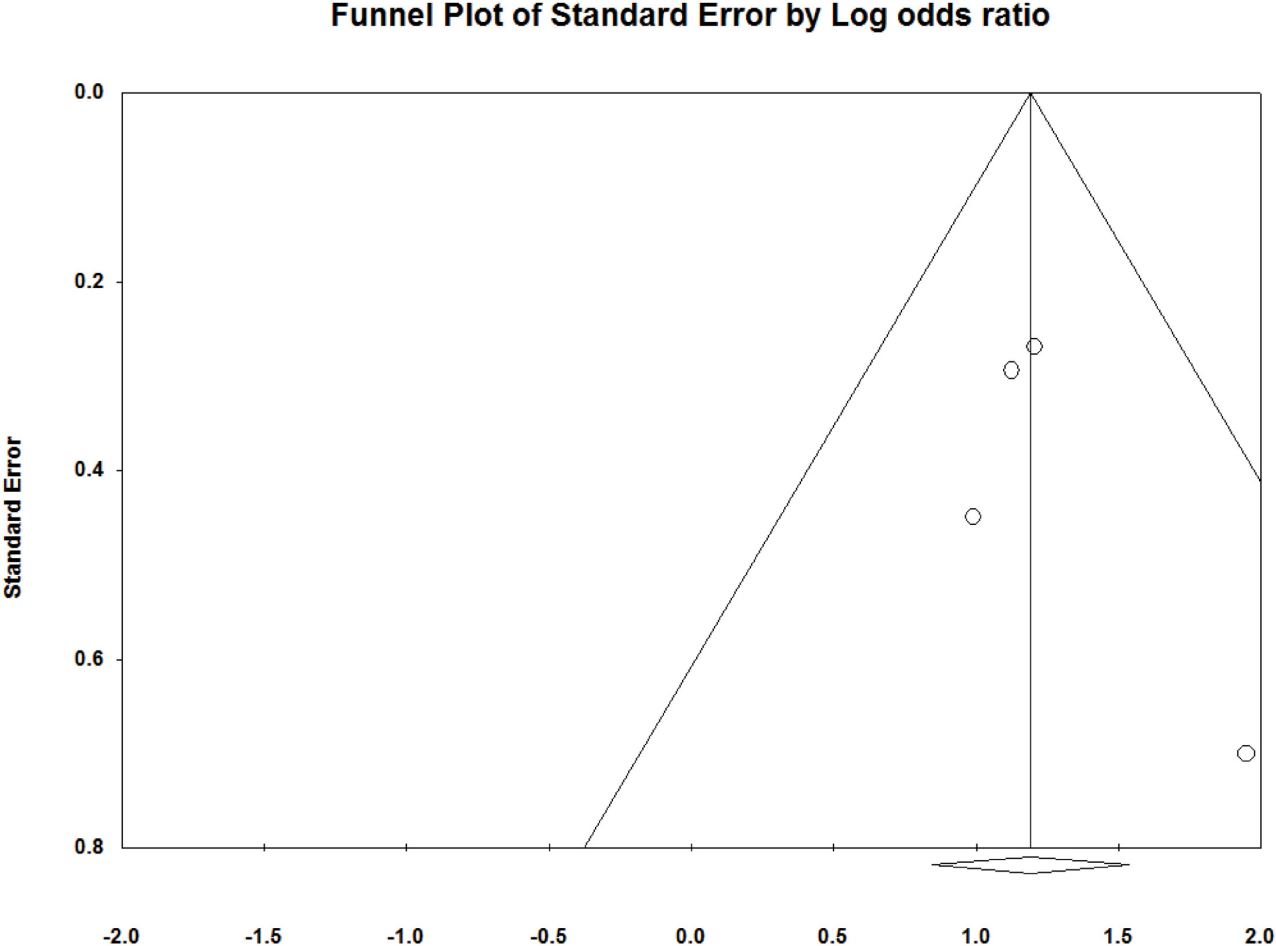
Funnel plot for publication bias in wound healing rate.

Funnel plots for the included studies are illustrated in Figs 5 and 6. These plots show little asymmetry, suggesting an absence of bias. Overall, we found no evidence of publication bias in this analysis.

## Discussion

This study identified four cohort studies reporting on 881 limbs, comparing the effect of angiosome-targeted and nonangiosome-targeted angioplasty to treat arterial occlusive disease in diabetic foot. Our meta-analysis showed that angiosome-targeted angioplasty resulted in an improved limb salvage rate (OR = 2.209, p = 0.001) and wound healing rate (OR = 3.290, p < 0.001) compared with nonangiosome-targeted angioplasty.

The improved outcomes seen in studies consistent with use of the angiosome concept compared with nonangiosome-targeted angioplasty could be explained by the absence of adequate collateral vessels. When adequate collateral vessels were present, the outcomes of nonangiosome procedures were comparable to those consistent with the angiosome concept [19]. However, collateral vessels in diabetic feet tend to be compromised [20, 21], suggesting that angiosome-targeted angioplasty should be preferentially used over nonangiosome angioplasty in diabetic foot.

Angioplasty in diabetic foot aims to prevent any major amputation, which correlates with a high mortality rate [10]. Hence, limb salvage may play an important role in improving longevity in diabetic foot patients with peripheral arterial disease. Several earlier studies have identified outstanding outcomes with respect to limb salvage employing angiosome-targeted angioplasty [15, 16, 20, 22]. Our results are consistent with those studies.

Ulcers in diabetic foot may reflect severe disease with considerable risk for ulcer chronicity that could lead to major amputation and death. Therefore, the threshold for performing revascularization in the diabetic foot should be lower than that for nondiabetics [16]. Diabetes produces a number of biomechanical, neuropathogenic, and immunogenic foot disorders [2, 17]. However, although most diabetic ulcers appear neuropathic, they have underlying ischemic components [23, 24]. Therefore, obtaining proper blood flow to the lesion is mandatory for the ulcer to heal [25]. As suggested in previous studies and our meta-analysis [15–17, 20, 22], providing direct blood flow to the specific area using the angiosome concept favorably affects ulcer healing in diabetic feet.

Of course, angioplasty employing the angiosome concept faces some difficult challenges. Diabetic patients with infrapopliteal atherosclerosis frequently develop concentric continuous vascular wall calcifications that could limit the effectiveness of endovascular angioplasty [20] and lead to revascularization of non-targeted vessels. In addition to that, when indirect revascularization is the only way to improve foot revascularization due to various causes, it needs to be done. Moreover, despite successful angioplasty, risks of delayed wound healing and major amputation remain [26, 27]. Complex interactions between atherosclerotic vessel disease and microvascular dysfunction in diabetic feet make the outcomes of angioplasty unpredictable [28].

Despite those limitations, previous studies and our meta-analysis have identified the effectiveness of angiosome-targeted angioplasty in managing diabetic foot, with a low revision rate that does not differ significantly from that of the nonangiosome group (OR = 0.747, p = 0.314). Therefore, when angiosome-targeted angioplasty is feasible for treating diabetic foot, it should be considered preferentially to nonangiosome-targeted angioplasty as a safe and effective treatment option.

This is the first meta-analysis of angiosome-targeted angioplasty in diabetic feet with peripheral arterial disease although there are some previous related systematic reviews [29, 30]. These previous studies suggested efficacy of direct revascularization in only lower limb ischemia not patients with diabetic feet. We identified that the diabetic foot has poor collateral vessel network so that angiosome-targeted angioplasty could be effective in only patients with diabetic feet. While its strength lies in our rigorous literature searches, there remain several limitations. Although NOS scores indicate the four studies to be of high quality, they were no randomized controlled studies. The study of presenting a meta-analysis shows only retrospective data and none of the studies were adequately powered, randomized, controlled trials comparing angiosome and non-angiosome groups. In addition, the absence of standardized direct and indirect angioplasty strategies in the included studies could be a critical source of bias. A further weakness in the studies was that wound definition was unclear in terms of location, infection status, and depth.

## Conclusion

Angiosome-targeted angioplasty in the treatment of diabetic feet has shown outstanding outcomes with respect to wound healing and limb salvage rate compared with nonangiosome-targeted angioplasty. Future large-scale and randomized studies with sufficient follow-up will further clarify the effectiveness of angiosome-targeted angioplasty in the management of diabetic foot.

## Supporting Information

S1 Fig List the 122 full-text excluded articles. (PDF)

S1 File PRISMA checklist. (DOC)
