# Supplementary material for: Is Angiosome-Targeted Angioplasty Effective for Limb Salvage and Wound Healing in Diabetic Foot? : A Meta-Analysis
Source: PLoS One. 2016 Jul 21;11(7):e0159523. doi: 10.1371/journal.pone.0159523 (PMC4956043; doi:10.1371/journal.pone.0159523)
Supplement: S1 Fig — (PDF) [file pone.0159523.s001.pdf]

List of 122 full-text excluded articles

Review (n = 16)

1. Revascularization for critical limb ischemia in diabetes: surgery or angioplasty? Tenna AS[1], Watson A, Stansby G. Angiology. 2014 Apr;65(4):272-3. doi: 10.1177/0003319713494465. Epub 2013 Jul 9.

2: Fährenkemper T, Klonek WM. [Therapeutic vascular surgery possibilities in diabetic foot syndrome]. Internist (Berl). 1999 Oct;40(10):1036-41. Review. German. PubMed PMID: 10541631.

3: Beyssen B, Pagny JY, Piquois A, Raynaud A, Sapoval M. [Critical limb ischaemia: endovascular treatment in diabetic patients?]. Arch Mal Coeur Vaiss. 2004 Dec;97 Spec No 3:33-9. Review. French. PubMed PMID: 15666480.

4: Mills JL Sr. Open bypass and endoluminal therapy: complementary techniques for revascularization in diabetic patients with critical limb ischaemia. Diabetes Metab Res Rev. 2008 May-Jun;24 Suppl 1:S34-9. doi: 10.1002/dmrr.829. Review. PubMed PMID: 18384110.

5: Alexandrescu V, Hubermont G. Primary infragenicular angioplasty for diabetic neuroischemic foot ulcers following the angiosome distribution: a new paradigm

for the vascular interventionist? Diabetes Metab Syndr Obes. 2011;4:327-36. doi: 10.2147/DMSO.S23471. Epub 2011 Aug 22. PubMed PMID: 21969804; PubMed Central PMCID: PMC3180522.

6: Alexandrescu V, Hubermont G. The challenging topic of diabetic foot revascularization: does the angiosome-guided angioplasty may improve outcome. J Cardiovasc Surg (Torino). 2012 Feb;53(1):3-12. Review. PubMed PMID: 22231524.

7: Peeters P, Verbist J, Keirse K, Callaert J, Deloose K, Bosiers M. Endovascular procedures and new insights in diabetic limb salvage. J Cardiovasc Surg (Torino). 2012 Feb;53(1):31-7. Review. PubMed PMID: 22231527.

8: Pedrajas FG, Cafasso DE, Schneider PA. Endovascular therapy: is it effective in the diabetic limb? Semin Vasc Surg. 2012 Jun;25(2):93-101. doi: 10.1053/j.semvascsurg.2012.04.006. Review. PubMed PMID: 22817859.

9: Georgakarakos E, Papanas N, Papadaki E, Georgiadis GS, Maltezos E, Lazarides MK. Endovascular treatment of critical ischemia in the diabetic foot: new thresholds, new anatomies. Angiology. 2013 Nov;64(8):583-91. doi: 10.1177/0003319712465172. Epub 2012 Nov 4. Review. PubMed PMID: 23129734.

10: Sumpio BE, Forsythe RO, Ziegler KR, van Baal JG, Lepantalo MJ, Hinchliffe RJ. Clinical implications of the angiosome model in peripheral vascular disease. J Vasc Surg. 2013 Sep;58(3):814-26. doi: 10.1016/j.jvs.2013.06.056. Review. PubMed PMID: 23972249.

11: Huang TY, Huang TS, Wang YC, Huang PF, Yu HC, Yeh CH. Direct Revascularization With the Angiosome Concept for Lower Limb Ischemia: A Systematic Review and Meta-Analysis. Medicine (Baltimore). 2015 Aug;94(34):e1427. doi: 10.1097/MD.0000000000001427. PubMed PMID: 26313796.

12: McCallum JC, Lane JS 3rd. Angiosome-directed revascularization for critical limb ischemia. Semin Vasc Surg. 2014 Mar;27(1):32-7. doi: 10.1053/j.semvascsurg.2014.11.003. Epub 2014 Nov 7. Review. PubMed PMID: 25812757.

13: Antonopoulos AG, Thomopoulos M, Trikas A. The role of the angiosome model in percutaneous intravascular and surgical reperfusion treatment of peripheral artery disease of the lower limbs. Hellenic J Cardiol. 2014 Jan-Feb;55(1):52-7. Review. PubMed PMID: 24491935.

14: Biancari F, Juvonen T. Angiosome-targeted lower limb revascularization for ischemic foot wounds: systematic review and meta-analysis. Eur J Vasc Endovasc

Surg. 2014 May;47(5):517-22. doi: 10.1016/j.ejvs.2013.12.010. Epub 2014 Jan 31. Review. PubMed PMID: 24491282.

15: Setacci C, Sirignano P, Galzerano G, Mazzitelli G, Sauro L, de Donato G, Benevento D, Cappelli A, Setacci F. Endovascular first as "preliminary approach" for critical limb ischemia and diabetic foot. J Cardiovasc Surg (Torino). 2013 Dec;54(6):679-84. Review. PubMed PMID: 24126506.

16: Albayati MA, Shearman CP. Peripheral arterial disease and bypass surgery in the diabetic lower limb. Med Clin North Am. 2013 Sep;97(5):821-34. doi: 10.1016/j.mcna.2013.03.009. Epub 2013 Apr 24. Review. PubMed PMID: 23992894.

Letter (n = 9)

1: Taylor GI, Palmer JH. 'Angiosome theory'. Br J Plast Surg. 1992 May-Jun;45(4):327-8. PubMed PMID: 1623352.

2: Alexandrescu VA. Commentary: Below-the-ankle subintimal angioplasty: how far can we push this application for lower limb preservation in diabetic patients? J Endovasc Ther. 2009 Oct;16(5):617-8. doi: 10.1583/09-2793C2.1. PubMed PMID: 19842732.

3: Bazan HA. Think of the angiosome concept when revascularizing the patient with critical limb ischemia. Catheter Cardiovasc Interv. 2010 May 1;75(6):837. doi: 10.1002/ccd.22572. PubMed PMID: 20432387.

4: Azuma N. Response to Commentary on 'Factors influencing wound healing of critical ischaemic foot after bypass surgery: is the angiosome important in selecting bypass target artery?'. Eur J Vasc Endovasc Surg. 2012 Jul;44(1):105. doi: 10.1016/j.ejvs.2012.04.003. Epub 2012 Apr 29. PubMed PMID: 22546642.

5: Azuma N. Response to letter to the editor: 'Factors influencing wound healing of critical ischaemic foot after bypass surgery: is the angiosome important in selecting bypass target artery?'. Eur J Vasc Endovasc Surg. 2013 Jan;45(1):99-100. doi: 10.1016/j.ejvs.2012.09.018. Epub 2012 Oct 30. PubMed PMID: 23116987.

6: Attinger C. Comment on: 'Factors influencing wound healing of critical ischaemic foot after bypass surgery: is the angiosome important in selecting bypass target artery?'. Eur J Vasc Endovasc Surg. 2013 Jan;45(1):99. doi: 10.1016/j.ejvs.2012.09.019. Epub 2012 Oct 30. PubMed PMID: 23116989.

7: Antoniou GA, Murray D, Antoniou SA, Kuhan G, Georgiadis GS. The angiosome-model as an effective paradigm to improve clinical outcomes of infra-popliteal revascularization. Int Angiol. 2013 Aug;32(4):443-5. Review.

PubMed PMID: 23822949.

8: Forsythe R, Hinchliffe R. Commentary on "Angiosome-targeted lower limb revascularization for ischaemic foot wounds: systematic review and meta-analysis". Eur J Vasc Endovasc Surg. 2014 May;47(5):523. doi: 10.1016/j.ejvs.2014.01.020. Epub 2014 Feb 24. PubMed PMID: 24573108.

9: Battal B, Celikkanat S, Karaman B, Akgun V. Re: percutaneous angioplasty in diabetic patients with critical limb ischemia. Korean J Radiol. 2014 Jan-Feb;15(1):178. doi: 10.3348/kjr.2014.15.1.178. Epub 2014 Jan 8. PubMed PMID: 24497811; PubMed Central PMCID: PMC3909854.

Case (n = 5)

1. Percutaneous transluminal angioplasty for critical limb ischemia in very elderly diabetic patients

2: Antono D. Percutaneous angioplasty in the diabetic foot. Acta Med Indones. 2007 Oct-Dec;39(4):192-3. PubMed PMID: 18046066.

3: Engelhardt M, Bruijnen H, Scharmer C, Wohlgemuth WA, Willy C, Wölfle KD. Prospective 2-years follow-up quality of life study after infrageniculate bypass

surgery for limb salvage: lasting improvements only in non-diabetic patients. Eur J Vasc Endovasc Surg. 2008 Jul;36(1):63-70. doi: 10.1016/j.ejvs.2008.01.026. Epub 2008 Mar 19. PubMed PMID: 18356087.

4: Gerassimidis T, Karkos CD, Karamanos D, Kamparoudis A. Current endovascular management of the ischaemic diabetic foot. Hippokratia. 2008 Apr;12(2):67-73. PubMed PMID: 18923656; PubMed Central PMCID: PMC2464305.

5: Gazzaruso C, Coppola A, Collaviti S, Saluzzo CM, Furlani F, Gallotti P, Clerissi J, Solerte BS, Giustina A. Percutaneous transluminal angioplasty for critical limb ischemia in very elderly diabetic patients. Aging Clin Exp Res. 2013 May;25(2):225-8. doi: 10.1007/s40520-013-0033-x. Epub 2013 Apr 18. PubMed PMID: 23739911.

Incomplete date (n = 61)

1: Lejay A, Georg Y, Tartaglia E, Gaertner S, Geny B, Thaveau F, Chakfe N. Long-term outcomes of direct and indirect below-the-knee open revascularization based on the angiosome concept in diabetic patients with critical limb ischemia. Ann Vasc Surg. 2014 May;28(4):983-9. doi: 10.1016/j.avsg.2013.08.026. Epub 2013 Dec 11. PubMed PMID: 24333196.

2. Clinical outcome of primary infrainguinal subintimal angioplasty in diabetic patients with critical lower limb ischemia

3. Clinical outcomes of lower limb peripheral vascular disease after endovascular intervention in patients with diabetes mellitus, critical limb ischemia and chronic kidney disease

4. Therapeutic effect of percutaneous transluminal angioplasty for infrapopliteal artery disease associated with critical limb ischemia in diabetic patients

5. The long-term outcomes of endovascular procedure in diabetic patients with critical limb ischaemia according to chronic kidney disease stage

6. Incidence of perioperative complications and its impact on clinical outcome after endovascular therapy for patients with diabetic ischemic foot presenting with isolated infrapopliteal lesions

7. Short-and long-term results of balloon angioplasty in diabetic patients with foot purulent-necrotic complications

8: Davies AH, Cole SE, Magee TR, Scott DJ, Baird RN, Horrocks M. The effect of diabetes mellitus on the outcome of angioplasty for lower limb ischaemia. Diabet Med. 1992 Jun;9(5):480-1. PubMed PMID: 1535305.

9: Durham JR, Horowitz JD, Wright JG, Smead WL. Percutaneous transluminal angioplasty of tibial arteries for limb salvage in the high-risk diabetic patient. Ann Vasc Surg. 1994 Jan;8(1):48-53. PubMed PMID: 8192999.

10: Karacagil S, Almgren B, Bowald S, Bergqvist D. Comparative analysis of patency, limb salvage and survival in diabetic and non-diabetic patients undergoing infrainguinal bypass surgery. Diabet Med. 1995 Jun;12(6):537-41. PubMed PMID: 7648829.

11: Faglia E, Favales F, Quarantiello A, Calia P, Brambilla G, Rampoldi A, Morabito A. Feasibility and effectiveness of peripheral percutaneous transluminal balloon angioplasty in diabetic subjects with foot ulcers. Diabetes Care. 1996 Nov;19(11):1261-4. PubMed PMID: 8908391.

12: Spence LD, Hartnell GG, Reinking G, Gibbons G, Pomposelli F, Clouse ME. Diabetic versus nondiabetic limb-threatening ischemia: outcome of percutaneous iliac intervention. AJR Am J Roentgenol. 1999 May;172(5):1335-41. PubMed PMID: 10227512.

13: Cavallini M, Caterino S, Murante G. Revascularization of the ischemic diabetic foot by popliteal-to-distal bypass. Minerva Cardioangiol. 1999 Jan-Feb;47(1-2):7-13. English, Italian. PubMed PMID: 10356936.

14: Isaksson L, Lundgren F. Prognostic factors for failure of primary patency within a year of bypass to the foot in patients with diabetes and critical ischaemia. Eur J Surg. 2000 Feb;166(2):123-8. PubMed PMID: 10724489.

15: Toursarkissian B, Hassoun HT, Smilanich RP, Godsey JB, Sykes MT. Efficacy of infrainguinal bypass for limb salvage in young diabetic patients. J Diabetes Complications. 2000 Sep-Oct;14(5):255-8. PubMed PMID: 11113687.

16: Panneton JM, Gloviczki P, Bower TC, Rhodes JM, Canton LG, Toomey BJ. Pedal bypass for limb salvage: impact of diabetes on long-term outcome. Ann Vasc Surg. 2000 Nov;14(6):640-7. PubMed PMID: 11128460.

17: Faglia E, Mantero M, Caminiti M, Caravaggi C, De Giglio R, Pritelli C, Clerici G, Fratino P, De Cata P, Dalla Paola L, Mariani G, Poli M, Settembrini PG, Sciangula L, Morabito A, Graziani L. Extensive use of peripheral angioplasty, particularly infrapopliteal, in the treatment of ischaemic diabetic foot ulcers: clinical results of a multicentric study of 221 consecutive diabetic subjects. J Intern Med. 2002 Sep;252(3):225-32. PubMed PMID: 12270002.

18: AhChong AK, Chiu KM, Wong MW, Hui HK, Yip AW. Diabetes and the outcome of infrainguinal bypass for critical limb ischaemia. ANZ J Surg. 2004 Mar;74(3):129-33. PubMed PMID: 14996159.

19: Lazaris AM, Tsiamis AC, Fishwick G, Bolia A, Bell PR. Clinical outcome of primary infrainguinal subintimal angioplasty in diabetic patients with critical lower limb ischemia. J Endovasc Ther. 2004 Aug;11(4):447-53. PubMed PMID:

15298514.

20: Faglia E, Dalla Paola L, Clerici G, Clerissi J, Graziani L, Fusaro M, Gabrielli L, Losa S, Stella A, Gargiulo M, Mantero M, Caminiti M, Ninkovic S, Curci V, Morabito A. Peripheral angioplasty as the first-choice revascularization procedure in diabetic patients with critical limb ischemia: prospective study of 993 consecutive patients hospitalized and followed between 1999 and 2003. Eur J Vasc Endovasc Surg. 2005 Jun;29(6):620-7. Epub 2005 Mar 28. PubMed PMID: 15878541.

21: Jacqueminet S, Hartemann-Heurtier A, Izzillo R, Cluzel P, Golmard JL, Ha Van G, Koskas F, Grimaldi A. Percutaneous transluminal angioplasty in severe diabetic foot ischemia: outcomes and prognostic factors. Diabetes Metab. 2005 Sep;31(4 Pt 1):370-5. PubMed PMID: 16369199.

22: Brannigan AE, Ahmad K, Tubassum M, Clarke Moloney M, Grace PA, Burke PE. Popliteal-to-distal artery bypass for diabetic limb salvage. Ir J Med Sci. 2006 Apr-Jun;175(2):28-31. PubMed PMID: 16872025.

23: Dick F, Diehm N, Galimanis A, Husmann M, Schmidli J, Baumgartner I. Surgical or endovascular revascularization in patients with critical limb ischemia: influence of diabetes mellitus on clinical outcome. J Vasc Surg. 2007 Apr;45(4):751-61. Epub 2007 Feb 15. PubMed PMID: 17306950.

24: Gu YQ, Zhang J, Qi LX, Yu HX, Li JX, Li XF, Guo LR, Luo T, Cui SJ, Wang ZG. Surgical treatment of 82 patients with diabetic lower limb ischemia by distal arterial bypass. Chin Med J (Engl). 2007 Jan 20;120(2):106-9. PubMed PMID: 17335650.

25: Faglia E, Clerici G, Clerissi J, Mantero M, Caminiti M, Quarantiello A, Curci V, Lupattelli T, Morabito A. When is a technically successful peripheral angioplasty effective in preventing above-the-ankle amputation in diabetic patients with critical limb ischaemia? Diabet Med. 2007 Aug;24(8):823-9. Epub 2007 Jun 8. PubMed PMID: 17559430.

26: Zhuang BX, Ma LB, Yu CL, Zhang T, Yang M, Shi B, Shi DZ. [Percutaneous transluminal angioplasty in treatment of lower limb peripheral arterial disease in diabetes patients: analysis of 150 cases]. Zhonghua Yi Xue Za Zhi. 2007 Jul 10;87(26):1821-4. Chinese. PubMed PMID: 17922990.

27: DeRubertis BG, Pierce M, Ryer EJ, Trocciola S, Kent KC, Faries PL. Reduced primary patency rate in diabetic patients after percutaneous intervention results from more frequent presentation with limb-threatening ischemia. J Vasc Surg. 2008 Jan;47(1):101-8. doi: 10.1016/j.jvs.2007.09.018. PubMed PMID: 18178459.

28: Malmstedt J, Leander K, Wahlberg E, Karlström L, Alfredsson L, Swedenborg J.

Outcome after leg bypass surgery for critical limb ischemia is poor in patients with diabetes: a population-based cohort study. Diabetes Care. 2008 May;31(5):887-92. doi: 10.2337/dc07-2424. Epub 2008 Feb 11. PubMed PMID: 18268064.

29: Alexandrescu VA, Hubermont G, Philips Y, Guillaumie B, Ngongang C, Vandenbossche P, Azdad K, Ledent G, Horion J. Selective primary angioplasty following an angiosome model of reperfusion in the treatment of Wagner 1-4 diabetic foot lesions: practice in a multidisciplinary diabetic limb service. J Endovasc Ther. 2008 Oct;15(5):580-93. doi: 10.1583/08-2460.1. PubMed PMID: 18840046.

30: Ferraresi R, Centola M, Ferlini M, Da Ros R, Caravaggi C, Assaloni R, Sganzaroli A, Pomidossi G, Bonanomi C, Danzi GB. Long-term outcomes after angioplasty of isolated, below-the-knee arteries in diabetic patients with critical limb ischaemia. Eur J Vasc Endovasc Surg. 2009 Mar;37(3):336-42. doi: 10.1016/j.ejvs.2008.12.001. Epub 2008 Dec 27. PubMed PMID: 19112033.

31: Kuusela J, Manninen HI, Karhapää P. Infrapopliteal balloon angioplasty for chronic critical limb ischemia in diabetic patients with uremia: when is it worth the effort? J Vasc Interv Radiol. 2009 Mar;20(3):342-6. doi: 10.1016/j.jvir.2008.11.022. Epub 2009 Jan 21. PubMed PMID: 19157896.

32: Neville RF, Attinger CE, Bulan EJ, Ducic I, Thomassen M, Sidawy AN. Revascularization of a specific angiosome for limb salvage: does the target artery matter? Ann Vasc Surg. 2009 May-Jun;23(3):367-73. doi: 10.1016/j.avsg.2008.08.022. Epub 2009 Jan 29. PubMed PMID: 19179041.

33: Alexandrescu V, Hubermont G, Philips Y, Guillaumie B, Ngongang Ch, Coessens V, Vandenbossche P, Coulon M, Ledent G, Donnay JC. Combined primary subintimal and endoluminal angioplasty for ischaemic inferior-limb ulcers in diabetic patients: 5-year practice in a multidisciplinary 'diabetic-foot' service. Eur J Vasc Endovasc Surg. 2009 Apr;37(4):448-56. doi: 10.1016/j.ejvs.2008.12.005. Epub 2009 Feb 11. PubMed PMID: 19213580.

34: Habib SH, Biswas KB, Akter S, Saha S, Ali L. Cost-effectiveness analysis of medical intervention in patients with early detection of diabetic foot in a tertiary care hospital in Bangladesh. J Diabetes Complications. 2010 Jul-Aug;24(4):259-64. doi: 10.1016/j.jdiacomp.2008.12.005. Epub 2009 Feb 23. PubMed PMID: 19231246.

35: Zhu YQ, Zhao JG, Liu F, Wang JB, Cheng YS, Li MH, Wang J, Li J. Subintimal angioplasty for below-the-ankle arterial occlusions in diabetic patients with chronic critical limb ischemia. J Endovasc Ther. 2009 Oct;16(5):604-12. doi: 10.1583/09-2793.1. PubMed PMID: 19842730.

36: Liistro F, Grotti S, Venturuzzo G, Angioli P, Turini F, Ducci K, Falsini G, Bellandi G, Ricci L, Anichini R, Bolognese L. [Clinical outcome of percutaneous revascularization by stent-assisted balloon angioplasty of femoro-popliteal and tibial vessels in diabetic patients with critical limb ischemia]. G Ital Cardiol (Rome). 2009 Nov-Dec;10(11-12):713-7. Italian. PubMed PMID: 20101897.

37: Hering J, Angelkort B, Keck N, Wilde J, Amann B. Long-term outcome of successful percutaneous transluminal angioplasty of the fibular artery in diabetic foot syndrome and single-vessel calf perfusion depends on doppler wave pattern at the forefoot. Vasa. 2010 Feb;39(1):67-75. doi: 10.1024/0301-1526/a000007. PubMed PMID: 20186678.

38: Iida O, Nanto S, Uematsu M, Ikeoka K, Okamoto S, Dohi T, Fujita M, Terashi H, Nagata S. Importance of the angiosome concept for endovascular therapy in patients with critical limb ischemia. Catheter Cardiovasc Interv. 2010 May 1;75(6):830-6. doi: 10.1002/ccd.22319. PubMed PMID: 20306500.

39: Wu YF, Gu YQ, Li XF, Yu HX, Qi LX, Guo LR, Cui SJ, Li JX, Zhang J, Wang ZG. [Preliminary evaluation of clinical effects of below-knee arterial bypass on diabetic lower limb ischemia]. Zhonghua Wai Ke Za Zhi. 2010 Feb 15;48(4):257-60. Chinese. PubMed PMID: 20388431.

40: Varela C, Acín F, de Haro J, Bleda S, Esparza L, March JR. The role of foot

collateral vessels on ulcer healing and limb salvage after successful endovascular and surgical distal procedures according to an angiosome model. Vasc Endovascular Surg. 2010 Nov;44(8):654-60. doi: 10.1177/1538574410376601. Epub 2010 Jul 30. PubMed PMID: 20675308.

41: Alexandrescu V, Ngongang C, Vincent G, Ledent G, Hubermont G. Deep calf veins arterialization for inferior limb preservation in diabetic patients with extended ischaemic wounds, unfit for direct arterial reconstruction: preliminary results according to an angiosome model of perfusion. Cardiovasc Revasc Med. 2011 Jan-Feb;12(1):10-9. doi: 10.1016/j.carrev.2009.12.002. Epub 2010 Oct 20. PubMed PMID: 21241966.

42: Kaputin MIu, Ovcharenko DV, Platonov SA, Chistiakov SP. Comparative analysis of remote results of transluminal balloon angioplasty in treatment of lower limb critical ischaemia in groups of patients with and without diabetes mellitus. Angiol Sosud Khir. 2010;16(3):41-6. English, Russian. PubMed PMID: 21275231.

43: Tan M, Pua U, Wong DE, Punamiya SJ, Chua GC, Teo N. Critical limb ischaemia in a diabetic population from an Asian Centre: angiographic pattern of disease and 3-year limb salvage rate with percutaneous angioplasty as first line of treatment. Biomed Imaging Interv J. 2010 Oct-Dec;6(4):e33. doi: 10.2349/biij.6.4.e33. Epub 2010 Oct 1. PubMed PMID: 21611069; PubMed Central PMCID: PMC3097802.

44: Claesson K, Kölbel T, Acosta S. Role of endovascular intervention in patients with diabetic foot ulcer and concomitant peripheral arterial disease. Int Angiol. 2011 Aug;30(4):349-58. PubMed PMID: 21747354.

45: Cardaioli P, Rigatelli G, Dell'avvocata F, Giordan M, Lisato G, Mollo F, Vassilev D, Nanjundappa A. Endovascular treatment of diabetic foot syndrome: results from a single center prospective registry using mixed coronary and peripheral techniques and equipment. J Interv Cardiol. 2011 Dec;24(6):562-8. doi: 10.1111/j.1540-8183.2011.00676.x. Epub 2011 Sep 12. PubMed PMID: 21910751

46: Iida O, Soga Y, Hirano K, Kawasaki D, Suzuki K, Miyashita Y, Terashi H, Uematsu M. Long-term results of direct and indirect endovascular revascularization based on the angiosome concept in patients with critical limb ischemia presenting with isolated below-the-knee lesions. J Vasc Surg. 2012 Feb;55(2):363-370.e5. doi: 10.1016/j.jvs.2011.08.014. Epub 2011 Nov 1. PubMed PMID: 22051875.

47: Ryu HM, Kim JS, Ko YG, Hong MK, Jang Y, Choi DH. Comparison of clinical outcome of infrapopliteal angioplasty between Korean diabetic and non-diabetic patients with critical limb ischemia. Circ J. 2012;76(2):335-41. Epub 2011 Nov 23. PubMed PMID: 22112858.

48: Azuma N, Uchida H, Kokubo T, Koya A, Akasaka N, Sasajima T. Factors influencing wound healing of critical ischaemic foot after bypass surgery: is the angiosome important in selecting bypass target artery? Eur J Vasc Endovasc Surg. 2012 Mar;43(3):322-8. doi: 10.1016/j.ejvs.2011.12.001. Epub 2012 Jan 9. PubMed PMID: 22237509.

49: Faglia E, Clerici G, Airoldi F, Tavano D, Caminiti M, Curci V, Mantero M, Morabito A, Edmonds M. Revascularization by angioplasty of type D femoropopliteal and long infrapopliteal lesion in diabetic patients with critical limb ischemia: are TASC II recommendations suitable? A population-based cohort study. Int J Low Extrem Wounds. 2012 Dec;11(4):277-85. doi: 10.1177/1534734612463701. Epub 2012 Oct 21. PubMed PMID: 23089965.

50: Matsuzaki K, Miyamoto A, Hakamata N, Fukuda M, Yamauchi Y, Akita T, Kuhara R, Tezuka S. Diabetic foot wounds in haemodialysis patients: 2-year outcome after percutaneous transluminal angioplasty and minor amputation. Int Wound J. 2012 Dec;9(6):693-700. doi: 10.1111/j.1742-481X.2012.01104.x. Epub 2012 Oct 24. PubMed PMID: 23095148.

51: Liistro F, Porto I, Angioli P, Grotti S, Ricci L, Ducci K, Falsini G, Ventoruzzo G, Turini F, Bellandi G, Bolognese L. Drug-eluting balloon in peripheral intervention for below the knee angioplasty evaluation (DEBATE-BTK): a randomized trial in diabetic patients with critical limb ischemia. Circulation.

2013 Aug 6;128(6):615-21. PubMed PMID: 23797811.

52: Troisi N, Ercolini L, Chisci E, Baggiore C, Chechi T, Manetti F, Del Pin B, Virgili R, Lepri GA, Landini G, Michelagnoli S. Diabetic Foot Infection: Preliminary Results of a Fast-Track Program with Early Endovascular Revascularization and Local Surgical Treatment. Ann Vasc Surg. 2015 Sep 11. pii: S0890-5096(15)00675-5. doi: 10.1016/j.avsg.2015.07.015. [Epub ahead of print] PubMed PMID: 26370745.

53: Shiraki T, Iida O, Takahara M, Soga Y, Yamauchi Y, Hirano K, Kawasaki D, Fujihara M, Utsunomiya M, Tazaki J, Yamaoka T, Shintani Y, Suematsu N, Suzuki K, Miyashita Y, Tsuchiya T, Uematsu M. Predictors of delayed wound healing after endovascular therapy of isolated infrapopliteal lesions underlying critical limb ischemia in patients with high prevalence of diabetes mellitus and hemodialysis. Eur J Vasc Endovasc Surg. 2015 May;49(5):565-73. doi: 10.1016/j.ejvs.2015.01.017. Epub 2015 Mar 4. PubMed PMID: 25747344.

54: Spillerova K, Biancari F, Leppäniemi A, Albäck A, Söderström M, Venermo M. Differential impact of bypass surgery and angioplasty on angiosome-targeted infrapopliteal revascularization. Eur J Vasc Endovasc Surg. 2015 Apr;49(4):412-9. doi: 10.1016/j.ejvs.2014.12.023. Epub 2015 Mar 5. PubMed PMID: 25747173.

55: Iida O, Takahara M, Soga Y, Yamauchi Y, Hirano K, Tazaki J, Yamaoka T,

Suematsu N, Suzuki K, Shintani Y, Miyashita Y, Uematsu M. Impact of angiosome-oriented revascularization on clinical outcomes in critical limb ischemia patients without concurrent wound infection and diabetes. J Endovasc Ther. 2014 Oct;21(5):607-15. doi: 10.1583/14-4692R.1. PubMed PMID: 25290786.

56: Skrepnek GH, Armstrong DG, Mills JL. Open bypass and endovascular procedures among diabetic foot ulcer cases in the United States from 2001 to 2010. J Vasc Surg. 2014 Nov;60(5):1255-64. doi: 10.1016/j.jvs.2014.04.071. Epub 2014 Jul 11. PubMed PMID: 25017514.

57: An JH, Jang YM, Song KH, Kim SK, Park SW, Jung HG, Kim DL. Outcome of percutaneous transluminal angioplasty in diabetic patients with critical limb ischaemia. Exp Clin Endocrinol Diabetes. 2014 Jan;122(1):50-4. doi: 10.1055/s-0033-1361102. Epub 2014 Jan 24. PubMed PMID: 24464598.

58: Houlind K, Christensen J, Hallenberg C, Jepsen JM. Early results from an angiosome-directed open surgical technique for venous arterialization in patients with critical lower limb ischemia. Diabet Foot Ankle. 2013 Dec 17;4. doi: 10.3402/dfa.v4i0.22713. eCollection 2013. PubMed PMID: 24358432; PubMed Central PMCID: PMC3867748.

59: Won KB, Chang HJ, Hong SJ, Ko YG, Hong MK, Jang Y, Choi D. Prognostic usefulness of metabolic syndrome compared with diabetes in Korean patients with

critical lower limb ischemia treated with percutaneous transluminal angioplasty.

Yonsei Med J. 2014 Jan;55(1):46-52. doi: 10.3349/ymj.2014.55.1.46. PubMed PMID:

24339286; PubMed Central PMCID: PMC3874905.

60: Serra R, Grande R, Scarcello E, Buffone G, de Franciscis S.

Angiosome-targeted revascularisation in diabetic foot ulcers. Int Wound J. 2015

Oct;12(5):555-8. doi: 10.1111/iwj.12162. Epub 2013 Oct 7. PubMed PMID: 24118695.

61: Iida O, Takahara M, Soga Y, Yamauchi Y, Hirano K, Tazaki J, Yamaoka T,

Suematsu N, Suzuki K, Shintani Y, Miyashita Y, Uematsu M. Worse limb prognosis

for indirect versus direct endovascular revascularization only in patients with

critical limb ischemia complicated with wound infection and diabetes mellitus.

Eur J Vasc Endovasc Surg. 2013 Nov;46(5):575-82. doi: 10.1016/j.ejvs.2013.08.002.

Epub 2013 Sep 11. PubMed PMID: 24034905.

abstract only (n = 31)

1. Endovascular revascularisation in type 2 diabetic patients with critical limb ischaemia: Comparison of direct and indirect revascularisarion according to angiosome model

2. Direct revascularization based on the angiosome model (AM) reduces risk of major amputations and increases life expectancy in diabetic patients with critical limb ischemia (CLI) and diabetic foot ulceration (DFU)

3. Clinical outcomes after endovascular revascularization in type 2 diabetic patients with critical

limb ischemia: Comparison of direct and indirect revascularization according to the angiosome model

4. Endovascular infrapopliteal interventions guided by an angiosome-based concept will improve clinical outcome in critical limb ischemia

5. Infrapopliteal bypass grafting for limb salvage in diabetic patients

6. Vascular assessment, surgery and the diabetic foot

7. Improved care of the diabetic foot: Promoting early intervention: 8th Annual One-Day Practical Diabetes International Foot Conference Commonwealth Conference Centre, London 7 December 2000

8. Endovascular treatment of critical limb ischemia in diabetic patients

9. Isolated lesions of infrapopliteal arteries in diabetic patients with critical limb ischemia: How effective is transluminal angioplasty?

10. Percutaneous angioplasty in the diabetic foot.

11. Balloon angioplasty of infrapopliteal artery in chronic lower limb ischemia with diabetes

12. Do the results of infragenual and infracrural bypass surgery justify the perioperative and postoperative complication rates? Risk profiles, morbidity, mortality and lower limb salvage in 156

patients, sub-group analysis for risk patients with diabetes mellitus and renal insufficiency .

13. Below-the-knee endovascular treatment in diabetic patients with critical limb ischemia in a single-center experience

14. Limb salvage and survival rate after endovascular treatment of infrapopliteal artery occlusion in diabetic patients with critical limb ischemia

15. Diabetic foot versus nondiabetic foot: Are there different outcomes following percutaneous transluminal angioplasty in patients with critical limb ischemia?

16. Below-the-knee endovascular treatment in diabetic patients with critical limb ischemia in a single-center experience: Midterm follow-up clinical results

17. Below the knee endovascular revascularisation strategy for limb salvage in diabetic patients

18 Limb salvage and survival rate after endovascular treatment of infrapopliteal artery occlusion in diabetic patients with critical limb ischemia

19. Percutaneous revascularisation of specific angiosome in critical limb ischaemia

20. Impact of angiosome in endovascular therapy on the limb salvage for the patients with critical limb ischemia presenting with isolated infrapopliteal lesions.

21. Angiosome directed angioplasty for limb salvage in critical limb ischemia

22. Vascular intervention in diabetic foot ulcer patients promotes healing and prevents amputation only when performed early after presentation of ulcer

23. Impact of the angiosome concept for endovascular therapy in patients with critical limb ischemia due to isolated below-the knee lesions

24. Study of the outcome of limb salvage angioplasty in high-risk patients with diabetic foot ulcer and limb ischaemia

25. Infrapopliteal angioplasty: Correlation between the treated vessel and injured angiosome

26. Strategies for infrapopliteal limb salvage intervention in the diabetic patient

27: Houlind K. [Angiosome-directed revascularization of critical limb ischaemia]. Ugeskr Laeger. 2015 Jul 27;177(31). pii: V03150264. Danish. PubMed PMID: 26238006.

28: Christensen J, Andersen PE. [Diabetic foot-artery intervention below the knee]. Ugeskr Laeger. 2013 Mar 18;175(12):795. Danish. PubMed PMID: 23582801.

29: Pierret C, Tourtier JP, Bordier L, Blin E, Duverger V. [Subintimal angioplasty and diabetic foot revascularisation]. Presse Med. 2011 Jan;40(1 Pt 1):10-6. doi: 10.1016/j.lpm.2010.09.005. Epub 2010 Oct 25. French. PubMed PMID:

20980123.

30. Direct flow utilizing the angiosome concept is valuable for salvaging limbs in critical limb ischemia patients

31: Lang W, Horch RE. [Distal extremity reconstruction for limb salvage in diabetic foot ulcers with pedal bypass, flap plasty and vacuum therapy]. Zentralbl Chir. 2006 Apr;131 Suppl 1:S146-50. German. PubMed PMID: 16575667.

List of 4 included articles

1: Alexandrescu V, Vincent G, Azdad K, Hubermont G, Ledent G, Ngongang C, ilimon AM. A reliable approach to diabetic neuroischemic foot wounds: below-the-knee angiosome-oriented angioplasty. J Endovasc Ther. 2011 Jun;18(3):376-87. doi: 10.1583/10-3260.1. PubMed PMID: 21679080.

2: Söderström M, Albäck A, Biancari F, Lappalainen K, Lepäntalo M, Venermo M. Angiosome-targeted infrapopliteal endovascular revascularization for treatment of diabetic foot ulcers. J Vasc Surg. 2013 Feb;57(2):427-35. doi: 10.1016/j.jvs.2012.07.057. Epub 2012 Dec 7. PubMed PMID: 23219512.

3: Acín F, Varela C, López de Maturana I, de Haro J, Bleda S, Rodriguez-Padilla

J. results of infrapopliteal endovascular procedures performed in diabetic patients with critical limb ischemia and tissue loss from the perspective of an angiosome-oriented revascularization strategy. Int J Vasc Med. 2014;2014:270539. doi: 10.1155/2014/270539. Epub 2014 Jan 6. PubMed PMID: 24527215; PubMed Central PMCID: PMC3914461.

4: Fossaceca R, Guzzardi G, Cerini P, Cusaro C, Stecco A, Parziale G, Perchinunno M, De Bonis M, Carriero A. Endovascular treatment of diabetic foot in a selected population of patients with below-the-knee disease: is the angiosome model effective? Cardiovasc Intervent Radiol. 2013 Jun;36(3):637-44. doi: 10.1007/s00270-012-0544-4. Epub 2013 Jan 29. PubMed PMID: 23358605.
